# Hypermethylation of EFEMP1 in the Hippocampus May Be Related to the Deficit in Spatial Memory of Rat Neonates Triggered by Repeated Administration of Propofol

**DOI:** 10.1155/2020/8851480

**Published:** 2020-12-19

**Authors:** Nu Zhang, Zhiyi Liao, Pinwen Wu, Hao Fang, Guoping Cai

**Affiliations:** ^1^Department of Anesthesiology, Jinshan Hospital, Fudan University, Shanghai 201508, China; ^2^Department of Anesthesiology, Minhang Hospital, Fudan University, Shanghai 201100, China; ^3^College of Information Engineering, Wuhan International Trade University, Wuhan 430205, China; ^4^Department of Anesthesiology, Zhongshan Hospital, Fudan University, Shanghai 200032, China; ^5^Department of Orthopedics, Jinshan Hospital, Fudan University, Shanghai 201508, China

## Abstract

It has been confirmed that repeated application of propofol, as an intravenous and short-fast-acting anesthetic, in neonatal animals or humans may produce long-term deficits in cognitive functions. With the aim of explaining the neurotoxic effects of repeated administration of propofol on neonatal rat pups from P7 to P9 especially from an epigenetic perspective, the present study used the Morris water maze to detect cognitive deficits in spatial learning and memory, Sequenom methylation on the CpG island located in the promoter region of epidermal growth factor-containing fibulin-like extracellular matrix protein 1 (EFEMP1) to assess the methylation level of this region, and Western blot to measure the expression of EFEMP1, TIMP-3, and MMP-9. As the results have shown, repeated propofol administration on neonatal rats caused significant systemic growth retardation, impairment of spatial learning and memory, and hypermethylation of the CpG sites in the promoter region of EFEMP1 accompanied by lower expression of EFEMP1 and TIMP-3 and enhanced expression of MMP-9. These data suggest that repeated propofol administration in neonatal rats may generate hypermethylation in the promoter region of EFEMP1 which results in downregulation of the expression of EFEMP1 and tissue inhibitor of metalloproteinase-3 (TIMP-3) but upregulation of the expression of matrix metalloproteinase-9 (MMP-9), which together may affect the stability of ECM to hamper the development of the central nervous system and therefore lead to deficits in cognitive functions.

## 1. Introduction

Though general anesthesia has its undoubted role in facilitating critical surgeries, still people started to consider its double-bladed actions on CNS since its principal mechanism is to blockade nervous traffic. Based on the current research, multiple or long-lasting anesthesia may cause some persistent neurotoxicity including deficits in memory and learning, at extreme ages [1], which is more often seen in patients with developing, immature brains. Moreover, in recent years, long-lasting cognitive deficits in children who received multiple or long-period anesthesia at their young age (<4 years old) have been found in several clinical retrospective studies [2] and plenty of animal pup experiments [3, 4].

As one type of often-used intravenous general anesthetics, propofol has been demonstrated to cause significant associated cognitive dysfunction at clinically relevant concentrations and durations [5]; however, the molecular mechanism of which still remains unclear. Many studies suggested that propofol may significantly increase neuroapoptosis in the rodent developing brain [6], which is likely related to neuroinflammation in the brain along with some other reasons [7]. Meanwhile, as far as we have known, extracellular matrix (ECM) molecules not only passively participate in the formation of the living environment for all cells inside our body but also play very active roles in the guidance of cellular proliferation, differentiation, and migration [8]. The development of the brain undergoes very complicated processes which are also led by various molecular signals including ECM molecules. Among various ECM molecules, epidermal growth factor-containing fibulin-like extracellular matrix protein 1 (EFEMP1), also known as fibulin-3, is a member of the fibulin glycoprotein family which shares an elongated structure with tandem arrays of calcium-binding epidermal growth factor- (EGF-) like domains and a carboxy-terminal fibulin-type module [9, 10]. Recent studies have shown that fibulin-3 is capable of upregulating tissue inhibitor of metalloproteinase- (TIMP-) 1 and TIMP-3, physically interacting with TIMP-3, and downregulating matrix metalloproteinase- (MMP-) 2, MMP-3, and MMP-9 [11, 12]. Matrix metalloproteinases (MMPs), as a family of protein-digesting enzymes, play important roles in maintenance and conversion of the ECM [13], and the activity and expression level of MMPs are strictly controlled by various mechanisms including transcription, activation of the precursor zymogens, and inhibition by the TIMP [14]. At present, four members of the TIMP family (TIMP-1 to TIMP-4) have been identified and three of them (TIMP1-3) have been identified to contribute to learning and memory [15, 16]. On the basis of our preliminary experiment, it was found that repeated propofol exposure caused hypermethylation of CpG sites in the promoter region of EFEMP1 and in turn reduction in expression of EFEMP1. Does reduced EFEMP1 expression have any effect on TIMP? And subsequently, could the activity and expression of MMPs also be affected to interfere with the stability of ECM in CNS, which therefore hampers the development of the brain? To solve these queries, we used mass spectrometry to measure and compare the methylation level of EFEMP1 and also assayed the alterations in the expression of EFEMP1 to explore whether the repeated application of propofol was capable of disturbing the stability of the living environment of neurons and thereby interfering with the development of the brain and long-term learning and memory.

## 2. Method and Materials

### 2.1. Animals

Twenty-two P3 (postnatal day 3) Sprague-Dawley male rat pups and their lactating dams (*n* = 4) were provided by Shanghai SLAC Laboratory Animal Co., Ltd. (China, SCXK (Shanghai) 2017-0005). Animals were raised in a laboratory animal room at specific pathogen-free (SPF) grade and fed with food and water ad arbitrium in 12 hr light/dark circulation. Pups were weighed every day and weaned on P21. All the treatments and experiments performed on the rats were approved by the Animal Welfare & Ethics Committee of Shanghai Public Health Clinical Laboratory, Shanghai, China.

### 2.2. Grouping and Drug Treatment

The 22 pups were randomly divided into three groups: control group (*n* = 7), vehicle group (*n* = 7), and propofol group (*n* = 8). The pups of the propofol group received intraperitoneal injections of 50 mg/kg propofol (Guangdong Jiabo Pharmaceutical Co., Ltd., Cat: 5C200112) for three consecutive days from P7 to P9 to investigate whether there is any effect of neonatal propofol exposure on their adult behavior. An equivalent volume of intraperitoneal injection of intralipid was used in the pups of the vehicle group. No interference was applied to the pups in the control group. During anesthesia, all pups were placed on a heating pad, and rectal temperature was maintained at 37 ± 1°C, and oxygen saturation was monitored using pulse oximetry and maintained at 95%. The pups were allowed to return to their dams after recovery, and all pups were placed by the flank of their mothers until ablactation occurred at P21. During this period, body weights were measured once a day since P3 and change of body weight was used as one indicator to reflect the growth of rat pups. Change of body weight was obtained by subtraction of the body weight of the day from that of the day prior to this day.

### 2.3. Morris Water Maze (MWM) Test

The Morris water maze test was performed for 5 days as previously described [17]. The water maze with a 120 cm diameter circular pool (Shanghai Xinruan Information Technology Company, Shanghai, China) was divided into four quadrants and enclosed with four different extramaze cues fixed at four quartiles of the pool periphery. A platform (5 cm in diameter) was placed 1 cm below the water surface in one quadrant. In the training sessions (P25-P28), all the rats were trained four times a day for four consecutive days and they were allowed to find the hidden platform within 120 s. The space probe trial was carried out at 8:00–12:00 on the 5th day (P29). The platform was removed, and both the latency time of each rat pup to locate the platform and the percentage of time they remained in the target quadrant were recorded [18].

### 2.4. Western Blot Analysis

Rats were sacrificed at P29. Then, the hippocampus was first homogenized with an ice-cold lysis buffer and then centrifuged at 12,000 rpm for 20 min at 4°C. Lastly, the supernatant was collected for protein content analysis. After quantitation of protein concentration via a BCA protein assay reagent, samples were separated by 10% SDS-PAGE gel, transferred to PVDF membranes under electrophoresis, and blocked with 5% bovine serum albumin for 2 hours. Subsequently, the membranes were incubated overnight at 4°C with the primary antibody for EFEMP1 (1 : 1000, ab106429, Abcam), TIMP-3 (1 : 1000, 5673, Cell Signaling Technology), and GAPDH (1 : 1000, 2118s, Cell Signaling Technology), then again incubated for another 2 hours with the HRP-conjugated secondary antibody. Immunoreactive proteins were detected using an enhanced chemiluminescence method, and the signal was normalized to GAPDH.

### 2.5. DNA Methylation Analyses

Genomic DNA was extracted from the hippocampus with the QIAamp DNA Mini Kit (Qiagen 51304, Germany) according to the manufacturer's instructions. The concentration and purity of the DNA were determined by absorbance at 260 and 280 nm. A total of 1.5 *μ*g genomic DNA from each sample was bisulfite-treated with the EZ DNA Methylation-Gold Kit (Cat: D5005, Zymo Research, USA). The Sequenom MassARRAY platform (CapitalBio, Beijing, China) [19] was used to perform the quantitative methylation analysis of EFEMP1 (GenBank accession number NC_005113). A detectable pattern is then analyzed for its methylation status. PCR primers were designed with EpiDesigner (http://www.epidesigner.com). For each reverse primer, an additional T7 promoter tag for in vivo transcription was added, as well as a 10-mer tag on the forward primer to adjust for melting temperature differences. We used the following primers on the basis of the reverse complemented strand of EFEMP1: 5′-aggaagagagTGTTTAATTTAATTTTGGATGGGTAG-3′and 3′-cagtaatacgactcactatagggagaaggctAAAAATAAACCAAATAAAACTCATCCT-5′. Mass spectra were obtained via MassARRAY Compact MALDI-TOF (Sequenom, San Diego, CA), and their methylation ratios (*n* = 4, each group) were generated using the EpiTYPER software version 1.0 (Sequenom, San Diego, CA).

### 2.6. Statistical Analysis

All data were expressed as mean ± standard deviation and analyzed with the Statistical Package for the Social Sciences (SPSS) version 15.0 (SPSS Inc., Chicago, IL, USA). Independent *t*-tests were performed to evaluate the significance of the difference in DNA methylation between the vehicle and propofol groups. Two-way ANOVA with tests of least significant difference (LSD) was used to assess the difference in body weight between groups. One-factor analysis of variance was used to evaluate the differences in rat behavior in the Morris water maze (MWM) test and expression of proteins in Western blot analysis. Differences were considered statistically significant if *p* < 0.05.

## 3. Results

### 3.1. Repeated Propofol Exposure from P7 to P9 Caused Less Change in Body Weight

**Figure 1:**
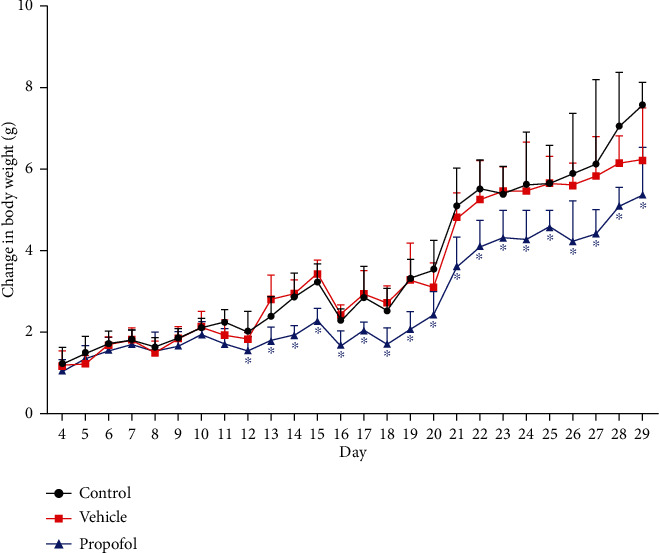
Alteration in weight from P4 to P29 after exposure to propofol (P7-P9). The body weight of animals at different measure time and related statistical data are shown in Table 1 in the supplementary material. ^∗^*p* < 0.05, versus the control group.

The change in body weight was obtained by subtraction of the weight of the day from the weight of the day immediately prior to the day. As shown in Figure 1, it was found that the change in pup weight of the propofol group was significantly less than that of the control and vehicle groups (*p* < 0.05) whereas there was no difference between the control group and the vehicle group (*p* > 0.05). All the related statistical data and parameters are shown in Table 1 in the supplementary material.

### 3.2. Repeated Propofol Exposure from P7 to P9 Caused Deficits in Spatial Learning and Memory

**Figure 2:**
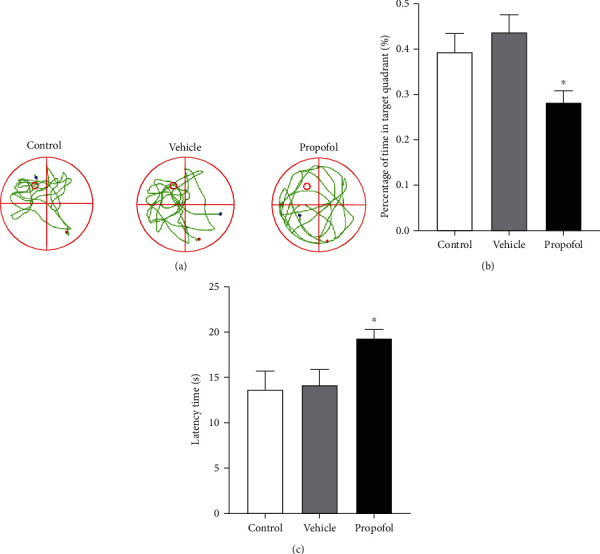
Effects of propofol on cognitive functions by the Morris water maze test. (a) Representative traces of rat movement in the open field test. The red circle in the left upper quadrant represented the position of the platform underwater, and the red dot represented the point from where rats were released into the water. (b) The percentage of time the rats remained in the target quadrant during the probe trial of the MWM test. (c) Escape latency for the rats to locate the platform during the probe trial of the MWM test. Related statistical data are shown in Table 2 in the supplementary material. ^∗^*p* < 0.05, versus the control group.

The Morris water maze test was performed on P29 to reflect the effects of propofol on long-term cognitive functions. As shown in Figure 2, the latent period for the rat pups in the propofol group was significantly longer than that in the control and vehicle groups (*p* < 0.05) while there was no difference between the control and vehicle groups (*p* > 0.05). Meanwhile, the percentage of the staying time in the target quadrant of the propofol group was obviously less than that of the control and vehicle groups (*p* < 0.05) whereas there was no difference between the control and vehicle groups (*p* > 0.05). All the related statistical data and parameters are shown in Table 2 in the supplementary material.

### 3.3. Propofol Caused Hypermethylation in the EFEMP1 Promoter Region

**Figure 3:**
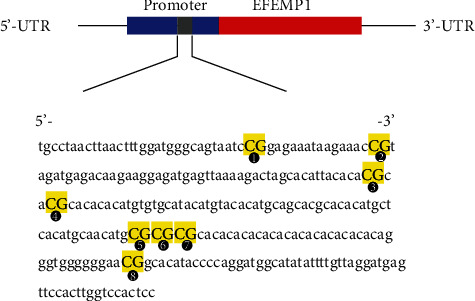
Schematic diagram of long interspersed nucleotide EFEMP1. EFEMP1 transcript consists of a 5′ untranslated region (5′-UTR) with internal promoter activity, an open reading frame, and a 3′-UTR. The sequence shown represented a 112-base pair fragment in the 5′-UTR of EFEMP1. PCR primers were designed based on the reverse complementary strands of this fragment. Numbers 1–8 refer to locations of the CpG site within the EFEMP1 elements tested, and the highlighted CpG units represented more than one CpG site detected at the same time.

**Figure 4:**
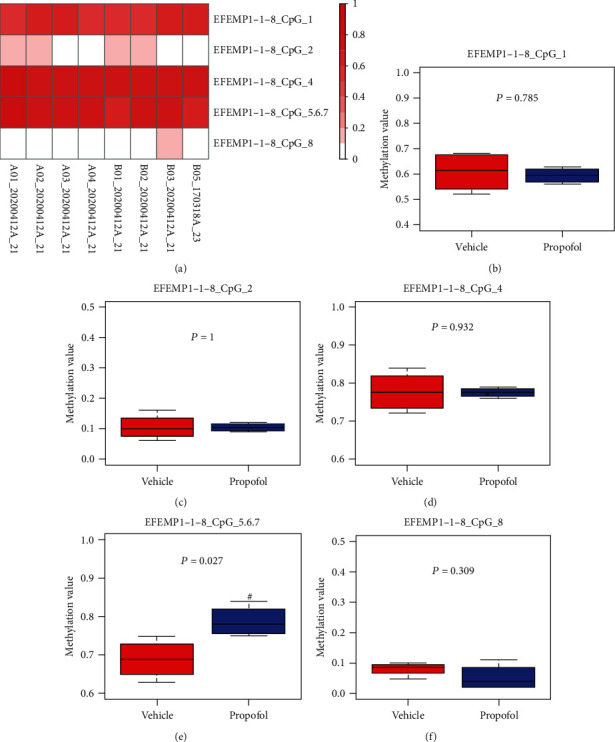
Comparison in the methylation levels of the CpG sites in the EFEMP1 promoter region between the vehicle group and the propofol group. (a) Was the heat map representing the methylation level of CpG sites in the EFEMP1 promoter region. Every row represented the methylation level of a CpG site while every line represented the methylation level of a sample. The discrepancy in the methylation level was displayed in red or white color. The more reddish the color, the higher the methylation level. (b–f) Were used to show the comparison in the methylation level of each CpG site in the EFEMP1 promoter region between the vehicle group and the propofol group. Due to missing data in the third CpG site, no result was obtained from it. ^#^*p* < 0.05, versus the vehicle group.

The EFEMP1 gene sequence was confirmed via GenBank, and a 112-base pair-long CpG island was found in the promoter region of EFEMP1. As shown in Figure 3, there were 8 CpG sites on this CpG island. Samples were analyzed by matrix-assisted laser desorption ionization time-of-flight mass spectrometry (MALDI-TOF-MS), which permits high-throughput identification of methylation sites and semiquantitative measurement at single or multiple CpG sites. The methylation level of each CpG site in the promoter region of EFEMP1 in the propofol and vehicle groups was assayed. As shown in Figures 4(b), 4(c), 4(d), and 4(f), there were no significant differences in the 1st, 2nd, 4th, and 8th CpG sites, respectively. Due to data missing in the 3rd CpG site, no result was acquired. As for the successive adjacent 5th, 6th, and 7th CpG sites, due to their close relationship, the methylation of these three CpG sites was detected as a whole, from which hypermethylation was observed in the propofol group (Figure 4(e)). The degree of methylation for every methylated site is shown in Table 3 in the supplementary material.

### 3.4. Effects of Repeated Propofol Exposure on Expression of EFEMP1, TIMP-3, and MMP-9 in the Hippocampus

**Figure 5:**
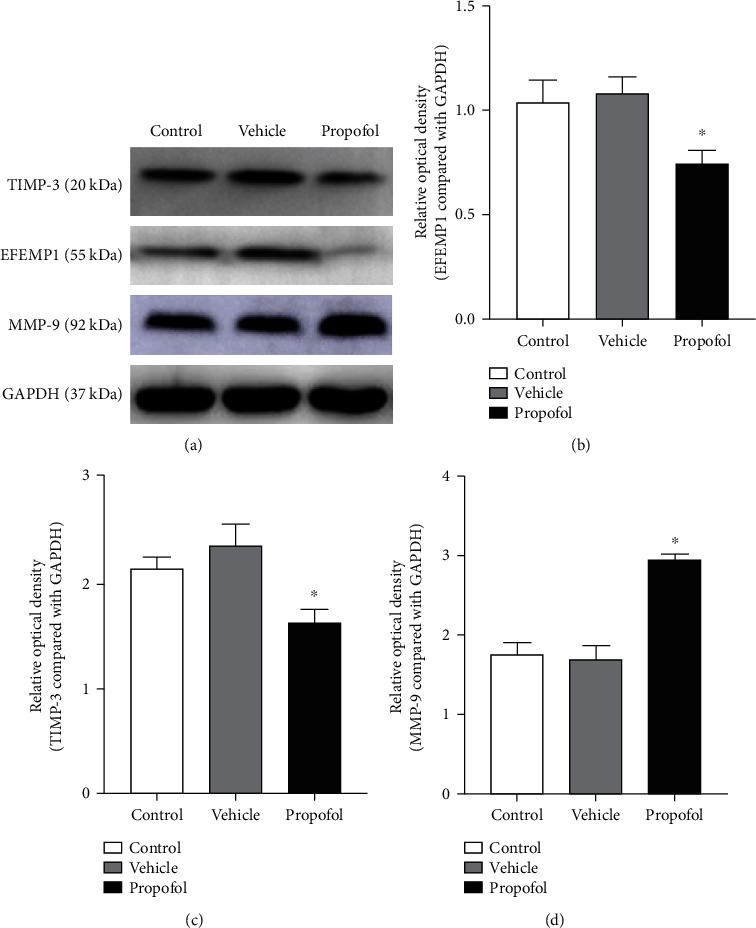
Alterations in the expression of EFEMP1 and TIMP-3 in the rat hippocampus. (a) The Western blotting results of EFEMP1, TIMP-3, and GAPDH. (b–d) Comparison in the levels of EFEMP1, TIMP-3, and MMP-9 by densitometry analysis among different groups. Related statistical data and parameters are shown in Table 4 in the supplementary material. ^∗^*p* < 0.05, versus the control group.

As displayed in Figures 5(b) and 5(c), there was a significantly lower expression of EFEMP1 and TIMP-3 in the propofol group than that in the control and vehicle groups (*p* < 0.05) whereas there was no meaningful difference between the control and vehicle groups (*p* > 0.05). Meantime, as shown in Figure 5(d), significantly enhanced expression of MMP-9 was observed in the propofol group than that in the control and vehicle groups (*p* < 0.05) while there was no meaningful difference between the control and vehicle groups (*p* > 0.05). Statistical data and parameters are shown in Table 4 in the supplementary material.

## 4. Discussion

Previous research on animals and human beings has confirmed that repeated/long-lasting/multiple application of general anesthesia (GA) may trigger neurotoxic changes in the developing, immature brain, which ultimately leads to a persistent deleterious impact on CNS, such as neuroapoptosis, disturbance in synaptogenesis, and cognitive deficits in spatial learning and memory [4]. More severely, the persistent harmful effects of GA mentioned above were more often observed in developing, immature, young brains (fetal, neonatal, or <4 years of age in human beings) rather than adult brains [3]. Propofol, as the fast-acting anesthetic, is not only used in adults but also widely used in pediatrics and obstetrics functioning via affecting GABA-A and NMDA receptors [20]. As revealed by our experiment, the rat pups after repeated exposure to propofol from P7 to P9 consecutively displayed impairment in spatial cognitive functions in the MWM test carried out on P29, which was consistent with several previous research studies [21, 22]. For the purpose of seeking a solution to relieve or avoid these unwanted side effects from propofol, a growing number of research studies have been carried out to explore the mechanism of propofol neurotoxicity.

Exposure to propofol at BGS may affect glial cells such as oligodendrocytes, the myelin sheath origin for the neurons in CNS, leading to the lack of myelination of neuronal axons and induction of further apoptosis of these unmyelinated neurons [6]. Meanwhile, administration of propofol at BGS may also alter the environmental status in CNS via suppressing neurotrophic factors such as BDNF and depolymerization of actin to interfere with the formation of microtubules and in turn adversely influencing axonal transport [23, 24]. And it is well known that epigenetic alterations are capable of influencing the expression of some specific genes and thereby affecting the production of the corresponding proteins [25]. Methylation/demethylation has been widely studied and regarded as one of the effective epigenetic manners to promote or hamper gene expression in many studies. Therefore, our research was aimed at exploring the mechanism of neurotoxicity caused by propofol from epigenetics.

In normal tissues, EFEMP1 is usually highly expressed by epithelial and endothelial cells, interacting with several other proteins of the extracellular matrix (ECM), contributing not only to the integrity of the basement membrane but also to the assembly of elastic fibers during embryonic development [9]. EFEMP1 is important to maintain cellular and tissue homeostasis, and its deregulation might lead to deregulated cell growth, invasion, and modification of ECM [26]. Meanwhile, ECM molecules are also critical for morphological changes of synapses between neurons in the brain which are involved in neural plasticity and learning and memory [27]. Moreover, EFEMP1 has been shown to stimulate the expression of tissue inhibitor of metalloproteinase- (TIMP-) 1 and TIMP-3 while inhibiting the expression of matrix metalloproteinase- (MMP-) 2, MMP-3, MMP-7, and MMP-9 [11, 28]. Sinner et al. have found that downregulation of EFEMP1 caused by hypermethylation of the EFEMP1 promoter region may affect metastasis of hepatocarcinoma (HCC) [29]. Moreover, EFEMP1 expression is also found to be regulated by promoter methylation in non-small-cell lung cancer (NSCLC) cells, and EFEMP1 could negatively modulate MMP-7 and MMP-2 [30]. It was found in our study that the hypermethylation detected in the promoter region of EFEMP1 in the rat pups after repeated exposure of propofol suggested the possibility that repeated administration of propofol may cause epigenetic modification of EFEMP1, therefore leading to the downregulation of EFEMP1 in the hippocampus and even the expression of some other downstream proteins demonstrated by Western blot.

MMP-9, as one pericellularly acting endopeptidase, regulates numerous cell processes and physiological functions via its role in remodeling the extracellular matrix [31, 32]. Plenty of studies confirmed that matrix metalloproteinases (MMPs) are substantial regulators of learning and memory and might be involved in neurodegeneration. Significant elevations in MMP-9 mRNA, protein levels, and enzymatic activity were also observed in the brain following a variety of stimuli [33, 34]. Studies on MMP-9 activation following learning in different behavioral tasks which all rely on activity in the hippocampus, such as the Morris water maze, inhibitory avoidance, contextual fear conditioning, object exploration, and response habituation, have demonstrated increases in the expression of pro and/or active forms of MMP-9 in the hippocampus [35, 36]. In line with these previous studies, as shown by our study, repeated application of propofol on neonatal rat pups caused the deficit in cognitive functions, which was proved by impairment of spatial memory via the MWM test accompanied with increased expression of MMP-9 in the hippocampus. Therefore, we postulated that the deficit in the long-term cognitive functions may be related to increased expression of MMP-9 in the hippocampus.

Hence, to explore the mechanism by which the expression of MMP-9 was elevated in the hippocampus on repeated exposure of propofol became the ultimate objective for our research. As displayed by previous studies, MMP activity is strictly regulated via various mechanisms including transcription, activation of the precursor zymogens, and inhibition by tissue inhibitor of metalloproteinase (TIMP) [13]. To date, four members of the TIMP family (TIMP-1 to TIMP-4) have been identified, among which TIMP-3, tightly bound to the ECM as well as both pro-MMP-2 and pro-MMP-9, is involved in cell proliferation, apoptosis, and angiogenesis [37]. Since TIMP-3 is a natural inhibitor of the proteolytic activity of MMP-9 and adamalysin proteins [38], the balance between MMP and TIMP controls ECM remodeling. Moreover, it was revealed that the MMP/TIMP system is responsible for the changes in neural activity in the central nervous system [39] and impaired cognitive dysfunction generated in TIMP-3 knockout mice due to deregulation of ECM homeostasis within the brain [16]. Consistent with these research studies, it was discovered in our study that the deficit in spatial memory in the 4-week-old rats after repeated treatment of propofol in the MWM test was accompanied by significantly reduced expression of TIMP-3 along with upregulation of MMP-9 in the hippocampus.

On the other hand, as for the functions of the pair of TIMP-3 and EFEMP1, there are several studies that can be cited below. Han et al. [11] observed a significant accumulation and expression overlap of both TIMP-3 and EFEMP1 between retinal pigment epithelia and Bruch's membrane in the eyes of ML and AMD patients. Additionally, Zayas-Santiago et al. discovered increased TIMP-3 along with decreased MMP-2 and MMP-9 in Bruch's membrane in EFEMP1^ki/ki^ mice. In summary, we inferred that EFEMP1 and TIMP-3 are positively related [10]. To put it more plainly, an increase in the expression of EFEMP1 causes a corresponding increase in the expression of TIMP-3 and vice versa. In our current study, repeated exposure of propofol from P7 to P9 may trigger hypermethylation in the promoter region of EFEMP1, which in turn caused the lower expression of EFEMP1, decreased expression of TIMP-3, and higher expression of MMP-9. The alterations in the expression of EFEMP1, TIMP-3, and MMP-9 ultimately led to impairment of cognitive functions in the 4-week-old rats. This suggests that we may improve the impairment of cognitive functions triggered by repeated propofol administration to neonatal rats by drugs to upregulate the expression of EFEMP1 and TIMP-3 or suppress the expression of MMP-9.

In conclusion, it may be hypothesized from our results that propofol administration in neonatal rats may generate hypermethylation in the promoter region of EFEMP1 which results in downregulation of the expression of EFEMP1 and TIMP-3 but upregulation of the expression of MMP-9 and subsequent neurotoxicity.

## Data Availability

Data included in this paper are available on request.

## Supplementary Materials

Supplementary Materials All the statistical data and parameters related to the statistical analysis in the current study are shown in the following four tables. Table 1: body weight of rats in different groups at different measure time. Table 2: data measured in the Morris water maze test. Table 3: methylation level of every CpG site. Table 4: expression of EFEMP1, TIMP-3, and MMP-9 in the hippocampus of rats.
