# Supplementary material for: Hypermethylation of EFEMP1 in the Hippocampus May Be Related to the Deficit in Spatial Memory of Rat Neonates Triggered by Repeated Administration of Propofol
Source: Biomed Res Int. 2020 Dec 19;2020:8851480. doi: 10.1155/2020/8851480 (PMC7765714; doi:10.1155/2020/8851480)
Supplement: Supplementary Materials — All the statistical data and parameters related to the statistical analysis in the current study are shown in the following four tables. Table 1: body weight of rats in different groups at different measure time. Table 2: data measured in the Morris water maze test. Table 3: methylation level of every CpG site. Table 4: expression of EFEMP1, TIMP-3, and MMP-9 in the hippocampus of rats. [file 8851480.f1.docx]

**The Supplementary Material：**

All the statistical data and parameters related to the statistical analysis in the current study were shown in the following four tables.

**Table 1** Body weight of rats in different groups at different measure timing

|  | Control group | Vehicle group | Propofol group | F value | p-value |
| --- | --- | --- | --- | --- | --- |
| 4th day | 1.24±0.15 | 1.17±0.14 | 1.07±0.09 | 0.50 | 0.612 |
| 5th day | 1.50±0.16 | 1.23±0.09 | 1.36±0.11 | 1.12 | 0.345 |
| 6th day | 1.74±0.11 | 1.70±0.08 | 1.56±0.11 | 0.89 | 0.426 |
| 7th day | 1.83±0.09 | 1.84±0.11 | 1.72±0.12 | 0.43 | 0.654 |
| 8th day | 1.64±0.09 | 1.50±0.11 | 1.53±0.06 | 0.70 | 0.511 |
| 9th day | 1.86±0.09 | 1.84±0.12 | 1.67±0.12 | 0.92 | 0.418 |
| 10th day | 2.11±0.09 | 2.13±0.15 | 1.94±0.11 | 0.82 | 0.455 |
| 11th day | 2.25±0.12 | 1.94±0.14 | 1.70±0.14 | 4.29 | 0.029 |
| 12th day | 2.03±0.19 | 1.83±0.04 | 1.55±0.12 | 3.57 | 0.048 |
| 13th day | 2.40±0.12 | 2.81±0.22 | 1.80±0.11 | 10.99 | 0.001 |
| 14th day | 2.88±0.22 | 2.95±0.14 | 1.93±0.09 | 14.07 | <0.001 |
| 15th day | 3.24±0.17 | 3.44±0.13 | 2.29±0.11 | 22.33 | <0.001 |
| 16th day | 2.30±0.10 | 2.43±0.09 | 1.69±0.12 | 13.68 | <0.001 |
| 17th day | 2.87±0.28 | 2.95±0.21 | 2.05±0.07 | 6.50 | 0.007 |
| 18th day | 2.53±0.21 | 2.73±0.16 | 1.71±0.15 | 10.49 | 0.001 |
| 19th day | 3.33±0.18 | 3.28±0.34 | 2.07±0.15 | 9.79 | 0.001 |
| 20th day | 3.56±0.27 | 3.13±0.18 | 2.43±0.20 | 7.13 | 0.005 |
| 21th day | 5.12±0.35 | 4.82±0.23 | 3.63±0.25 | 8.23 | 0.003 |
| 22th day | 5.52±0.27 | 5.27±0.36 | 4.12±0.22 | 7.11 | 0.005 |
| 23th day | 4.40±0.26 | 4.48±0.21 | 3.33±0.24 | 7.73 | 0.003 |
| 24th day | 5.63±0.49 | 5.47±0.45 | 4.28±0.25 | 3.57 | 0.048 |
| 25th day | 5.66±0.35 | 5.66±0.25 | 4.58±0.15 | 6.21 | 0.008 |
| 26th day | 5.90±0.56 | 5.61±0.20 | 4.23±0.35 | 5.28 | 0.015 |
| 27th day | 6.13±0.79 | 5.85±0.35 | 4.42±0.21 | 3.60 | 0.047 |
| 28th day | 7.06±0.49 | 6.14±0.26 | 5.11±0.16 | 9.31 | 0.002 |
| 29th day | 5.58±0.21 | 6.23±0.49 | 4.38±0.41 | 5.99 | 0.010 |

**Table 2** Data measured in Morris water maze test

|  |  | Control  group | Vehicle  group | Propofol  group | F value | p-value |
| --- | --- | --- | --- | --- | --- | --- |
| Percentage of time in target quadrant of MWM test | | 0.39±0.04 | 0.44±0.04 | 0.28±0.03 | 5.07 | 0.017 |
| latent of finding the platform in MWM test | | 13.60±2.14 | 14.17±1.71 | 19.33±0.94 | 3.96 | 0.037 |

**Table 3** Methylation level of every CpG site

| CpG site | Vehicle group | Propofol group | t value | p-value |
| --- | --- | --- | --- | --- |
| 1 | 60.75% | 59.50% | 0.29 | 0.785 |
| 2 | 10.50% | 10.50% | 0.00 | 1.000 |
| 3 | NANE | NANE | NANE | NANE |
| 4 | 77.75% | 77.50% | 0.09 | 0.932 |
| 5、6、7 | 78.75% | 69.00% | 2.95 | 0.027 |
| 8 | 8.00% | 5.25% | 1.15 | 0.309 |

**Table 4** Expression of EFEMP1, TIMP3 and MMP9 in the hippocampus of rats

|  | Control group | Vehicle group | Propofol group | F value | p-value |
| --- | --- | --- | --- | --- | --- |
| TIMP3 | 2.12±0.11 | 2.34±0.20 | 1.61±0.13 | 5.94 | 0.013 |
| EFEMP1 | 1.04±0.10 | 1.08±0.08 | 0.74±0.07 | 4.60 | 0.028 |
| MMP9 | 1.76±0.14 | 1.71±0.16 | 2.14±0.15 | 28.73 | <0.001 |
